# Multiple Fibroids of the Uterus

**Published:** 1901-04-13

**Authors:** 


### MULTIPLE FIBROIDS OF THE UTERUS.

Dr. Bland Sutton[1] relates a case of profuse bleeding due to fibroids, in which, on removing the uterus, he found within it four sessile fibroids projecting into its cavity, and so moulded together as to form facets on their contact surfaces such as are found in multiple gall stones. These fibroids were about the size of pigeons' eggs. The cut surface of the uterine wall was very thickly dotted with small rounded fibroids, and on carefully hardening the specimen in methylated spirit, and making complete sections, it was found to contain a multitude of fibroids, varying in size from a coriander seed to a cherry stone. From a careful computation of the tumours in the various sections this uterus, which scarcely exceeded the dimensions of a fist, contained 120 fibroids. An interesting feature in these minute growths was the similarity they bore to the large fully-developed tumours; each was globular, and on section quite white, so that the contrast in colour with the red uterine muscle fibre made them conspicuous objects on the cut surface. Each fibroid, too, was sharply differentiated from the uterine tissue by a thin capsule. In all that were examined the tumour cells were found disposed around the blood vessels, and this arrangement was so obvious that the tumours might be described as bundles of plain muscle fibres twined round and intimately associated with the walls of the capillaries. Indeed the relation of the vessels to the cell elements of these seedling tumours was such as to suggest that the muscular coats of the vessels were the source of the fibroids.

[1] Brit. Med. Jour., April 6

